# Correction: SARS-CoV-2 suppression depending on the pH of graphene oxide nanosheets

**DOI:** 10.1039/d3na90044d

**Published:** 2023-05-17

**Authors:** Md. Saidul Islam, Masahiro Fukuda, Md. Jakir Hossain, Nurun Nahar Rabin, Ryuta Tagawa, Mami Nagashima, Kenji Sadamasu, Kazuhisa Yoshimura, Yoshihiro Sekine, Terumasa Ikeda, Shinya Hayami

**Affiliations:** a Department of Chemistry, Faculty of Advanced Science and Technology, Kumamoto University 2-39-1 Kurokami Kumamoto 860-8555 Japan hayami@kumamoto-u.ac.jp; b Institute of Industrial Nanomaterials, Kumamoto University 2-39-1 Kurokami, Chuo-ku Kumamoto 860-8555 Japan; c Division of Molecular Virology and Genetics, Joint Research Center for Human Retrovirus Infection, Kumamoto University 2-2-1 Honjo Kumamoto 860-0811 Japan; d Graduate School of Medical Sciences, Kumamoto University Kumamoto 860-0811 Japan; e Tokyo Metropolitan Institute of Public Health Tokyo 169-0073 Japan; f Priority Organization for Innovation and Excellence, Kumamoto University 2-39-1 Kurokami, Chuo-ku Kumamoto 860-8555 Japan; g International Research Center for Agricultural and Environmental Biology (IRCAEB) 2-39-1 Kurokami, Chuo-ku Kumamoto 860-8555 Japan

## Abstract

Correction for ‘SARS-CoV-2 suppression depending on the pH of graphene oxide nanosheets’ by Md. Saidul Islam *et al.*, *Nanoscale Adv.*, 2023, https://doi.org/10.1039/D3NA00084B.

The authors regret that the affiliation of the author Terumasa Ikeda was incorrectly listed in the original manuscript. The correct affiliation is listed above. The affiliation 'e' was incompletely listed in the original manuscript. The correct affiliation is given below. The authors also regret that the following funding grants were not attributed to the correct funders. The correct funders are listed below:

JPMJTM20SL: Japan Science and Technology Agency (JST)

JP17H01200 and 22K07103: Japan Society for the Promotion of Science (JSPS)

The Royal Society of Chemistry apologises for these errors and any consequent inconvenience to authors and readers.

## Supplementary Material

